# Ability of ambulatory ECG-based T-wave alternans to modify risk assessment of cardiac events: a systematic review

**DOI:** 10.1186/1471-2261-14-198

**Published:** 2014-12-20

**Authors:** Xiao-Qing Quan, Hong-Lian Zhou, Lei Ruan, Jia-Gao Lv, Ji-Hua Yao, Feng Yao, Kui Huang, Cun-Tai Zhang

**Affiliations:** Department of Geriatrics, Tongji Hospital, Tongji Medical College, Huazhong University of Science and Technology, Wuhan, 430030 China; Department of Cardiology, Tongji Hospital, Tongji Medical College, Huazhong University of Science and Technology, Wuhan, 430030 China; Department of Cardiology, Tongji ShenZhi Hospital, Wuhan, China

**Keywords:** T-wave alternans, Ambulatory electrocardiogram, Sudden cardiac death, Cardiac event

## Abstract

**Background:**

Exercise-based spectral T-wave alternans (TWA) has been proposed as a noninvasive tool-identifying patients at risk of sudden cardiac death (SCD) and cardiac mortality. Prior studies have indicated that ambulatory electrocardiogram (AECG)-based TWA is an important alternative platform to exercise for risk stratification of cardiac events. This study sought to review data regarding 24-hour AECG-based TWA and to discuss its potential role in risk stratification of fatal cardiac events across a series of patient risk profiles.

**Methods:**

Prospective clinical studies of the predictive value of AECG-based TWA obtained with daily activity published between January 1990 and November 2014 were retrieved. Major endpoints included composite endpoint of SCD, cardiac mortality, and severe arrhythmic events.

**Results:**

Data were accumulated from 5 studies involving a total of 1,588 patients, including 317 positive and 1,271 negative TWA results. Compared with the negative group, positive group showed increased rates of SCD (hazard ratio [HR]: 7.49, 95% confidence interval [CI]: 2.65 to 21.15), cardiac mortality (HR: 4.75, 95% CI: 0.42 to 53.55), and composite endpoint (SCD, cardiac mortality, and severe arrhythmic events, HR: 5.94, 95% CI: 1.80 to 19.63). For the 4 studies evaluating TWA measured using the modified moving average method, the HR associated with a positive versus negative TWA result was 9.51 (95% CI: 4.99 to 18.11) for the composite endpoint.

**Conclusions:**

The positive group of AECG-based TWA has a nearly six-fold risk of severe outcomes compared with the negative group. Therefore, AECG-based TWA provides an accurate means of predicting fatal cardiac events.

**Electronic supplementary material:**

The online version of this article (doi:10.1186/1471-2261-14-198) contains supplementary material, which is available to authorized users.

## Background

The T-wave alternans (TWA) phenomenon, a repeating ABABAB pattern in the morphology and amplitude of the ST-segment or T-wave, has long been recognized and linked to arrhythmogenesis [1–3]. TWA has been proposed as noninvasive means of stratifying patients for the risk of sudden cardiac death (SCD) and cardiac mortality [1, 4, 5].

TWA value can be obtained during exercise testing (bicycle or treadmill) or AECG recording [1, 2, 5]. Exercise-based TWA has been mainly analyzed by frequency-domain spectral method [1, 2, 4, 6]. A target heart rate range of 105 to 110 beats/min for a sustained period (>2 min) was determined for microvolt TWA assessed during exercise testing [1, 6]. Therefore, not all patients can undergo a spectral TWA test, for example, patients taking medications such as beta-blockers, digoxin, and those with physical limitations. Recent study by Jackson et al. showed that spectral method was not widely applicable in patients hospitalized with acute decompensated heart failure, 49% were ineligible for spectral TWA testing, due either to physical inability to undertake the test, AF, or pacemaker dependency [7]. In another study by Tapanainen et al., of the 379 consecutive post-acute myocardial infarction (AMI) patient population, TWA could be analyzed reliably during exercise test in only 53% of the cases [8].

Because of inability to achieve the target heart rate, the presence of excessive ectopy, nonsustained TWA, or excessive signal noise due to motion or respiration, there is a relatively high incidence (19% to 46% of all cases) of indeterminate test results [1, 7, 9–11]. For the prognostic value of indeterminate TWA results, the results was controversial. Some studies have suggested that an indeterminate TWA tests due to patient factors was a strong predictor of SCD or cardiac arrest [8, 10, 12], but conflicting opinions exists [7, 9, 13, 14].

Ambulatory electrocardiogram (AECG) recording-based TWA represents an important alternative platform to stratify patients at risk for serious cardiac events [1, 2]. TWA testing based on 24-hour Holter ECG recorded during daily activity does not require heart rate elevation and so overcomes some of the limitations of spectral method [5, 15–17]. Prior studies with AECG-based TWA analysis have shown that an abnormal TWA result is modestly associated with cardiac events and that a normal result has a high negative predictive value [18–21]. However, negative studies have also appeared, including two prospective studies in patients with chronic heart failure [22] and AMI [23].

We therefore performed this systematic review and meta-analysis to determine the utility of 24-hour Holter AECG-based TWA for risk stratification of cardiac events in a wide variety of patient populations.

## Methods

### Ethics statement

The Ethics Committee (IRB) of Tongji Hospital (Wuhan City, Hubei Province, China) recognizes that this systematic review and meta-analysis of de-identified, publicly available data does not constitute “human subjects research” as defined by relevant national regulations, and therefore does not require Ethics Committee review.

### Search criteria

A systematic review of the available literature was performed according to the PRISMA (preferred reporting items for systematic reviews and meta-analyses) guidelines (Additional file 1). This article evaluates the potential value of 24-hour Holter AECG recording-based TWA in risk stratification of fatal cardiac events in a wide variety of patient populations. We performed a systematic literature search for prospective studies published in English between January 1990 and November 2014. The search was restricted to full-text English publications and human subjects. Using the terms "T wave alternans", "ambulatory electrocardiogram (ECG)", "Holter", we searched the following databases: MEDLINE, EMBASE, Cochrane Library, International Network of Agencies for Health Technology Assessment, and the Web of Science. A second search of articles published by authors identified in the initial search and a review of the bibliographies of all articles were performed to identify additional articles for review. We also hand-searched reviews and previous meta-analysis for additional potentially relevant studies.

We excluded review articles, abstracts, book chapters, conference proceedings, and correspondence. If more than one study reported on the same cohort, then we only used the most recent publication of that cohort. Articles of subcohorts were excluded if an article using the entire cohort was previously included. When the same research study was found in multiple journals or repositories, only one instance of the study was included.

### Inclusion criteria

Studies were included if they met the following criteria: 1) prospective clinical study of ≥ 100 human subjects; 2) 24-hour Holter AECG-based TWA obtained with daily activity; 3) reported meaningful clinical endpoints including SCD, cardiac mortality, and/or ventricular arrhythmias; 4) provided clear definition of positive or negative TWA. Duplicate publications and studies with a follow-up of less than 6 months were excluded.

Two investigators (Q.X. and Z.H.) independently reviewed the titles and abstracts of these articles and excluded those that clearly did not meet the inclusion criteria. A consensus was reached on which articles should be completely reviewed for potential inclusion in this study.

### Data extraction

Two investigators (Q.X. and Z.H.) independently reviewed potential articles blinded to the author, journal, and institution. Data for each article were abstracted, including study design, inclusion and exclusion criteria, clinical characteristics of participants, details regarding TWA result, end points of the study, duration of follow-up, raw data when provided, and reported findings, including hazard ratios (HR).

### Quality analysis of study

We assessed study quality based on the following assessment of the articles [9]: 1) the follow-up completed was beyond 90% of the cohort, 2) adjudication of outcomes was blinded to the results of TWA testing, and 3) a multivariate analysis using other standard predictors of cardiac events was performed. A study was considered at least of fair quality if it fulfilled the first criteria. A study was considered good quality if one of the other two criteria was met. This quality assessment was not used to assess study inclusion or exclusion, but was included as a study characteristic.

### Statistical analysis

The outcomes of each study were presented as HR with confidence interval (CI) of TWA for the prediction of SCD, cardiac mortality, or severe arrhythmic events at follow-up. After demonstration with Cochran’s Q test and I^2^ statistic of the homogeneity of the results, we used a random effects model developed by DerSimonian and Laird [24]. Publication bias was evaluated with the use of Begg’s test. Finally, we performed sensitivity analyses to examine the influence of each study on the pooled estimate by omitting each study one at a time. All statistical tests were 2-sided and were evaluated at a significance level of 0.05. STATA version 12.0 (College Station, TX, USA) was used to conduct all analyses.

## Results

### Study characteristics

**Figure 1:**
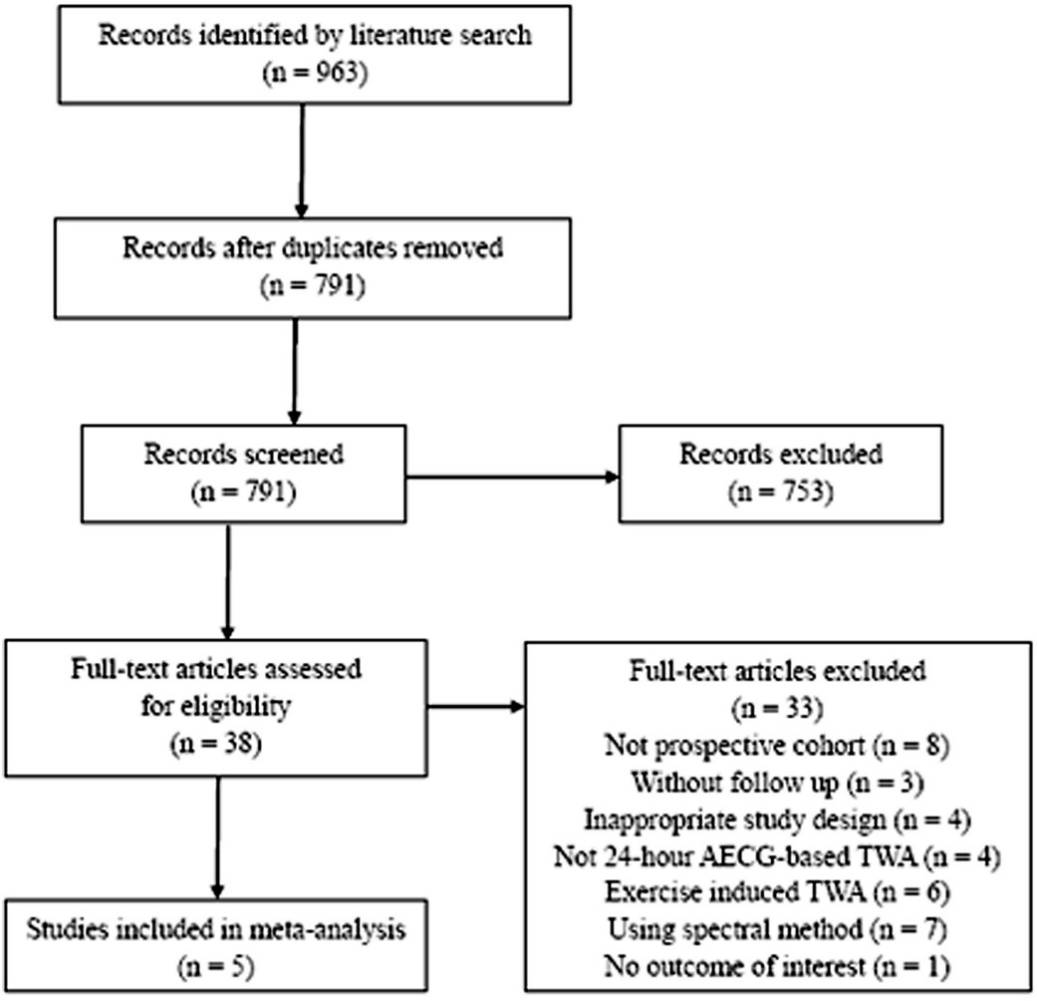
**PRISMA flow diagram.** Summary of the evidence search and selection

An initial literature search using the previously mentioned search terms identified 963 potential articles for inclusion in this study. After reviewing and eliminating clearly ineligible studies, we identified 5 prospective studies involving a total of 1,588 participants (Figure 1) [18–22]. One other study did not provide enough primary data [23], an attempt was made to contact the author, but failed to receive the original data. Therefore, this study was not included in our current analysis. All studies were identified in MEDLINE, although some were also found in other databases. All studies were classified as being of good quality.

### Predictive accuracy of AECG-based TWA

The baseline characteristics of included studies, stratified by the study of origin, are presented in Table 1. The studies enrolled an overall number of 1,588 patients who were followed up for an average of 33 months. Overall, mean age was 64 years, 74% of patients were men, and approximately 72% of patients had underlying ischemic heart disease. A positive TWA result observed overall in 317 (20%) of the patients (Table 2), 87 (28%) patients reached the endpoint. Whereas a negative result observed in 1,271 (80%) of the patients (Table 2), 120 (9%) patients reached the endpoint.

**Table 1:** **Characteristics of prospective cohort studies of AECG-based TWA**

Study	Total (n)	Mean age (yrs)	Men (%)	ICD implanted (%)	Population	Mean LVEF (%)	Average follow-up (months)
Sakaki et al. 2009 [19]	295	66	72	7	LVEF < 40%, dilated left ventricle	34	13
Sulimov et al. 2012 [20]	111	64	76	-	Post-MI	46.6	12
Yu et al. 2012 [21]	219	56	81	0	Acute MI	>35%^*^	16
Monasterio et al. 2012 [22]	650	63	71	-	symptomatic CHF corresponding to NYHA classes II and III	<35%^†^	48
Hoshida et al. 2013 [18]	313	70	74	1	Post-MI	47	39

*Abbreviations: AECG* ambulatory electrocardiogram, *CHF* chronic heart failure, *LVEF* left ventricular ejection fraction, *MI* myocardial infarction, *NYHA* New York Heart Association, *-* not stated. ^*^Ninety-two percent patients had LVEF greater than 35%. ^†^Fifty-five percent patients had LVEF smaller than 35%.

**Table 2:** **Test characteristics of AECG-based TWA**

Study	End Point	HR (95% CI)	P value	TWA Pos %	TWA Neg %	Cut-off value (μV)	Lead	Method
Sakaki et al. 2009 [19]	Cardiac mortality^*^	17.1 (6.3–46.6)	P <0.0001	18	82	65	V1 or V5	MMA
Sulimov et al. 2012 [20]	SCD	5.01 (1.5–17.0)	0.005	41	59	53.5	-	MMA
Yu et al. 2012 [21]	SCD	15.07 (2.88-78.68)	0.0031	22	88	47	V2 or V5	MMA
Monasterio et al. 2012 [22]	Cardiac mortality	1.44 (0.97–2.13)	0.068	24	76	3	-	LLR
Hoshida et al. 2013 [18]	Fatal arrhythmic events	5.8 (1.6–20.8)	0.0072	5	95	64	V1 or V5	MMA

*Abbreviations: AECG* ambulatory electrocardiogram, *HR* hazard ratio, *MMA* modified moving average, *Neg* negative, *Pos* positive, *SCD* sudden cardiac death, *-* not stated. ^*^One patient who received ICD defibrillation therapy was included.

In the study by Monasterio et al. (Table 3) [22], for the end point of cardiac mortality, the prognostic value of TWA was negative (HR: 1.44, 95% CI: 0.97–2.13). For the end point of SCD, the prognostic value of TWA was positive (HR: 2.29, 95% CI: 1.31–4.00).

**Table 3:** **Combined HR of AECG-based TWA related to fatal cardiac events by endpoint and study method**

Group	No. of studies	HR (95% CI)
Composite endpoint: SCD, cardiac mortality, and severe arrhythmic events	5	5.94 (1.80 to 19.63)
Endpoint: SCD	2	7.49 (2.65 to 21.15)
Sulimov et al.		
Yu et al.		
Endpoint: Cardiac mortality	2	4.75 (0.42 to 53.55)
Sakaki et al.		
Monasterio et al.		
Studies using MMA method	4	9.51 (4.99 to 18.11)
Sakaki et al.		
Sulimov et al.		
Yu et al.		
Hoshida et al.		

*Abbreviations: AECG* ambulatory electrocardiogram, *HR* hazard ratio, *MMA* modified moving average, *SCD* sudden cardiac death.

**Figure 2:**
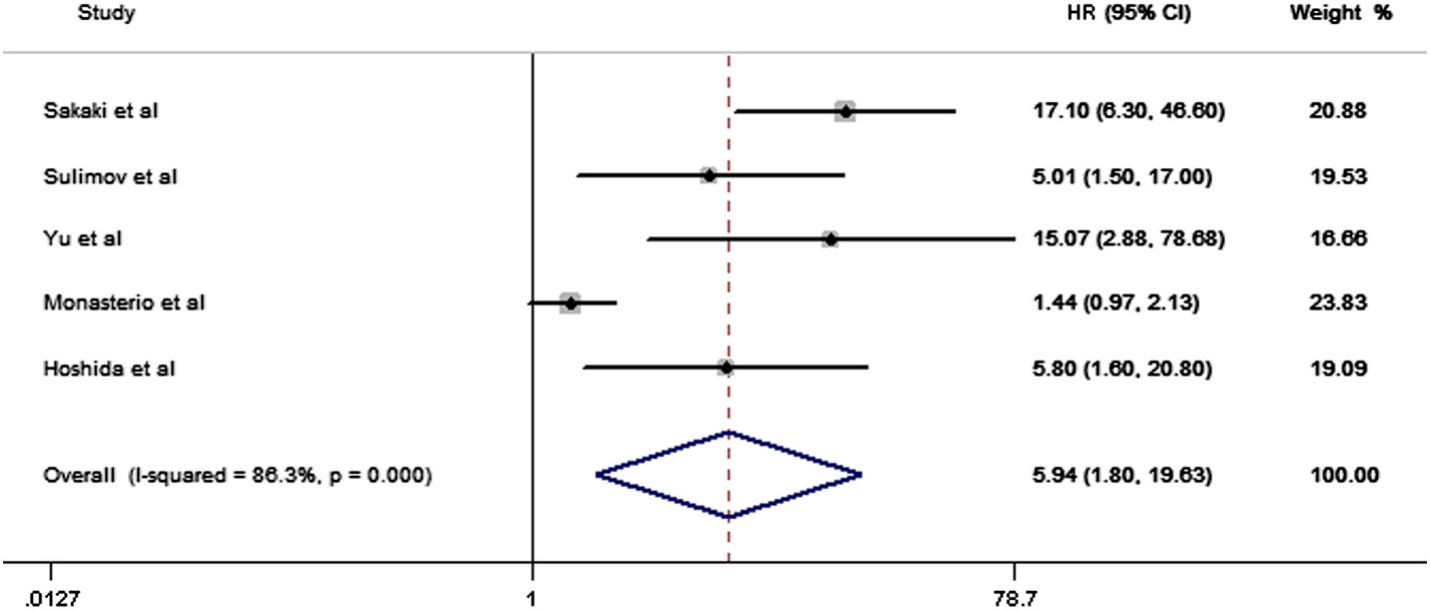
**Random-effects meta-analysis of prospective cohort studies that examined AECG-based TWA and fatal cardiac events.**

We pooled data across studies to investigate the predictive ability of TWA (Tables 2 and 3). In our primary analysis of the composite endpoint of SCD, cardiac mortality, and severe arrhythmic events, the risk of patients with positive TWA results was significantly increased compared to negative results (HR: 5.94, 95% CI: 1.80 to 19.63) (Figure 2, Table 3). Subgroup summary estimates showed that TWA-positive outcome was associated with increased risk of SCD (HR: 7.49, 95% CI: 2.65 to 21.15) and cardiac mortality (HR: 4.75, 95% CI: 0.42 to 53.55) (Table 3). Four studies evaluating TWA measured using the modified moving average (MMA) method, MMA-TWA-positive outcome was significantly associated with the composite endpoint (SCD, cardiac mortality, and severe arrhythmic events, HR: 9.51, 95% CI: 4.99 to 18.11).

## Discussion

We conducted the first meta-analysis of 24-hour AECG-based TWA as a predictor of cardiac events in a wide variety of patient populations, accumulating prospective data on more than 1,500 subjects. Overall, we have found that a positive TWA result predicted nearly six-fold risk of composite endpoint (SCD, cardiac mortality, and severe arrhythmic events) compared with a negative result.

As for the study that didn’t provide HR [23], 199 patients (mean age was 62 years, mean LVEF was 45%) with AMI undergoing 24-hour Holter ECG-based TWA assessment using the modified moving average (MMA) method. The primary outcome was sudden death and sustained ventricular arrhythmia treated with external electric cardioversion during 6-month follow-up period. TWA was positive in 8.5% patients (cut-off value was 46 μV). Survival analyses using Cox survival models revealed that TWA was not significantly associated with the incidence of the primary outcome (P = 0.15).

Studies with exercise-based and AECG-based TWA have shown that an abnormal TWA test indicates an increased risk for arrhythmic events or mortality [1–3, 5]. Studies with the exercise-based TWA is more extensive [1, 2]. However, measurement of exercise-based TWA using spectral method requires a specialized protocol and a target heart rate range of 105 to 110 beats/min for >2 min [1, 6]. As a result, spectral method has limited applicability in patients with heart failure or undertake beta-blocking agents. For studies with high ineligible rate (49% to 53%) of spectral method-based TWA test results in decompensated heart failure [7] and post-AMI [8] patients, the incomplete TWA test was the most significant predictor of mortality. Moreover, of the patients able to perform the test, TWA did not predict mortality in both studies [7, 8]. Alternative strategies would be required for the significant proportion of ineligible patients.

Furthermore, spectral method has a relatively high incidence (19% to 46%) of indeterminate test results [1, 7, 9–11], rising debating on the interpretation of TWA test results. The interpretation of indeterminate TWA in the individual patient must be integrated with the clinical history of that patient [1, 9]. Recordings with respiration, muscle, or other motion artifacts or electrode noise provide no prognostic information [11]. Inconsistency remains in current practice as to whether an indeterminate TWA test should be considered abnormal [7–10, 12–14].

Unlike the spectral method, AECG recording-based TWA could be analyzed in 24-hour Holter recording with daily activity, risk stratification is based on the peak TWA value, which permits visual examination to verify the presence and magnitude of TWA [15, 25]. AECG recording-based TWA does not require elevation of heart rate to a target level, it affords an opportunity to assess cardiac electrical instability in patients who cannot exercise or taking beta-blocking agents [5, 16, 17]. Indeterminate results are infrequent (3% to 5%) as no target heart rate is needed [2]. It remains important to use AECG-based TWA to risk stratify the patients who are ineligible for exercise-induced TWA test or have an indeterminate result. Furthermore, AECG analysis allows patients to be monitored for TWA during daily activity, incorporates the influences of heightened sympathetic nerve activity, disturbed nighttime breathing, and episodes of intense physiological or mental stress, which cannot be replicated during exercise stress tests.

Of the 5 included studies, 4 studies used the MMA method [18–21], one study used the Laplacian likelihood ratio (LLR) method [22]. Recently, Orini et al. performed a simulation study in vivo in humans to compare the accuracy of four commonly used methodologies, including spectral, MMA and LLR methods, for T-wave alternans mapping in electrograms [26]. They found that all methodologies provided accurate electrogram-TWA estimation/detection in ideal conditions, while LLR was the most accurate and robust, providing better detection-rates in noisy conditions.

MMA is the most used method employed for measures of AECG-based TWA in clinical studies [1, 15]. There are two standard cut-points for MMA: 47 μV, which indicates “abnormal” and 60 μV which indicates “severely abnormal” test results [1]. Recently, it has become possible to measure AECG-based TWA amplitude precisely [5]. It provides an opportunity for physicians to use TWA magnitude in risk assessment.

Previously, a meta-analysis performed by Rizas et al. shows that TWA using MMA method is powerful predictor of mortality in post-MI patients (HR: 5.53, 95% CI: 3.14 to 9.72) [27]. For the four clinical studies included in their study, there are both case-control and retrospective studies. Furthermore, TWA has been measured based on exercise or 24-hour AECG. There are several strengths of our meta-analysis of TWA. First, all of the studies analyzed the TWA based on the 24-hour AECG. Second, we assessed the predictive value of AECG-based TWA in a wide variety of patient populations, including both ischemic and non-ischemic cardiomyopathy with a wide range of LVEF. Finally, in contrast to many earlier TWA studies, we used SCD, cardiac mortality, and severe arrhythmic events, rather than all-cause mortality, as the endpoint.

### Study limitations

There are several limitations to this study. First, all data were derived from prospective cohort studies of AECG-based TWA. We did not identify any randomized studies meeting our inclusion criteria. Second, the 5 included studies varied in study design, patient characteristics and duration of follow-up. Nevertheless, there was no statistical evidence of heterogeneity. Third, the end points of the individual studies used in the summary calculations were slightly different (e.g., cardiac mortality vs. SCD vs. fatal arrhythmic events) and may have introduced some inconsistency when comparing across studies. Finally, there were not sufficient data contained in the included studies to determine the incremental prognostic value of AECG-based TWA independent of other predictors of arrhythmic events, such as heart rate variability, heart rate turbulence, baroreflex sensitivity, or LVEF. Although AECG-based TWA seems clinically useful in this meta-analysis, the additional prognostic value of AECG-based TWA when used with other predictors of risk stratification is unclear.

## Conclusions

We studied the use of AECG-based TWA as a predictor of cardiac events in a meta-analysis of more than 1,500 subjects. We found that the positive TWA result predicted a nearly six-fold risk of a cardiac event compared with the negative result. Given the challenge of risk stratification for fatal cardiac events that faces today’s cardiologist and the economic implications for today’s healthcare system, our study helps understand the value and the limitations of AECG-based TWA.

## Electronic supplementary material

Additional file 1: **PRISMA 2009 Checklist.** (DOC 63 KB)
